# Case of an American Girl, Deaf, Dumb, and Blind

**Published:** 1818-04

**Authors:** 


*For the London Medical and Physical Journal.*

### *Case of an American Girl, deaf, dumb, and blind.* [From the Boston Intelligencer.]

IF proofs of vigorous intellect in the deaf and dumb are admirable, how much more wonderful are evidences of a similar nature in persons who suffer the additional misfortune of being blind!

I heard a benevolent lady mention the name of Julia Brace, a girl about eleven years old, living in the vicinity of Hartford, who is afflicted with the triple calamity of blindness, deafness, and dumbness, having lost the senses of sight and hearing, by the violence of a typhus fever, at the age of four years. On visiting her myself, I learned that the extreme poverty and the obscurity of her parents, have prevented her from being known or particularly noticed, except by the charitable ladies of the town, and a few gentlemen, who have been induced by motives of curiosity to examine her conduct. The following facts and little anecdotes I relate for your amusement.

Her form and features are regular and well proportioned. Her temper is mild and affectionate. She is much attached to her infant sister, often passes her hand over the mouth and eyes* of the child, in order to ascertain whether it is crying, and soothes its little distresses with all the assiduity and success of a talkative or musical nurse. All objects which she can readily handle, she applies to her lips, and rarely fails of determining their character. If any thing is too large for examination in this way, she makes her fingers the interpreters of the texture and properties, and is seldom mistaken. She will beat apples or other fruit from the tree, and select the best with as much judgment as if she possessed the faculty of sight. She often wanders in the field, and gathers flowers, to which she is directed by the pleasantness of their odour. Her sense of smelling is remarkably exquisite, and appears to be an assistant guide with her fingers and lips.

A gentleman one day gave her a small fan. She inquired of her lips what it was; and, on being informed, returned it to the gentleman's pocket. The mother observed, that Julia already possessed one fan: she probably thought, that another would be superfluous. The gentleman gave the same fan to a neighbouring girl, whom Julia was in the habit of visiting. She went a few days after, to visit her companion, whose toys she passed under the review of her fingers and lips; and, among other things, the fan, the identity of which she instantly discovered, and again restored to the pocket of the gentleman, who happened to be present.

She feels and admires mantle-piece ornaments, and never breaks or injures the most brittle furniture, even in a strange room.

She is as obedient as other children in general. The jar of her mother's foot upon the floor effectually prevents the

---

* Probably to discover whether the mouth be distorted, or there are any tears on the cheek.

commission of a fault: but she easily distinguishes the stamping of one of the children from that of her mother, and obeys or not, as she pleases.

Her parents, as you may well suppose, have not been able to indulge her in dress; but, when she receives articles of clothing, or ornaments, as presents, she is highly gratified to find, that they resemble in form and fashion those of her playmate. She has, as you perceive, a spice of female vanity! At a tea-table, she behaves with more gentility than many a miss who has the benefit of eyes, by which to adjust her motions and attitudes.

A gentleman once made several experiments, with a view to satisfy himself whether she really had the discernment which she was reported to possess. Among other arts for effecting his object, he pretended to carry away her infant sister. She immediately detected the cheat, by ascertaining that his umbrella remained upon the table. She then went out of the door, and picked the head of a large thistle in full bloom, brought it in, smelling of it as she came, and offered it to the gentleman, apparently as a nosegay. He reached out his hand; but, instead of giving it, she archly pricked his hand, by way of retort for his freedom in testing her sagacity.

